# Refractory autoimmune thrombocytopenia in an infant with a de novo TLR7 gain-of-function variant

**DOI:** 10.1007/s10875-024-01824-4

**Published:** 2024-10-21

**Authors:** Surabhi Menon, Diane Maurice, Lauren A. Robinson, Joshua Milner, Virginia Pascual, Carola G. Vinuesa, Shipra Kaicker

**Affiliations:** 1 New York Presbyterian Weill Cornell Medical College, New York, US; 2 Memorial Sloan Kettering, New York, US; 3 Francis Crick Institute, London, UK; 4 Gale and Ira Drukier Institute for Children's Health, Weill Cornell Medical College, New York, US; 5 Hospital for Special Surgery, New York, US; 6 Institute of Genomic Medicine, Columbia University, New York, US

To the Editor:

Toll-like receptor 7 (TLR7) is an endosomal pattern recognition receptor that recognizes single-stranded RNA (ssRNA). TLR7 ligation activates downstream inflammatory pathways, including NF-kB and Type I Interferon (IFN). While TLR7 plays a vital role in anti-viral immunity, its overactivity has been linked to B cell-driven autoimmunity in mice and humans [1]. Gain-of-function (GOF) variants in TLR7, located on the X chromosome, have been recently described in pediatric patients with SLE and neuroinflammation and can be inherited. [2–4] Here we describe a 2-year-old developmentally normal female with severe refractory immune thrombocytopenia (ITP) following an adenoviral infection at 8 months of age. She was found to carry a de novo, novel heterozygous missense TLR7 G818V variant that we show confers GOF activity. Unlike the other TLR7GOF entities, ITP remains the sole clinical and laboratory manifestation.

The proband was healthy until the age of 8 months when she presented to the emergency department with sudden onset generalized petechiae and bruising. Her examination was otherwise unremarkable. She had no lymphadenopathy or hepatosplenomegaly. Her initial complete blood count revealed an isolated severe thrombocytopenia (platelet count= 2000/microliter) and a clinical diagnosis of Immune Thrombocytopenia (ITP) was made. Baseline quantitative immunoglobulins including IgG levels and lymphocyte subsets were normal. There was a mild elevation in CD3-positive T lymphocytes (see Supplemental Materials for detailed clinical and laboratory data). She was admitted and started on first-line ITP-directed therapy with 2 doses of Intravenous Immunoglobulin (IVIG) with no response. Over the next few days, she also developed a mild Coombs-negative non-hemolytic anemia not explained by any significant clinical bleeding. Aside from minimal blood-streaked emesis and stool positive for occult blood, no other significant source of blood loss was found. Additional work up for a cause for anemia was negative.

Due to her young age and bi-lineage cytopenias, a bone marrow aspiration and biopsy was performed to rule out a hematological malignancy before a steroid trial. The bone marrow evaluation showed hypercellular bone marrow with erythroid and megakaryocytic hyperplasia and many hematogones, consistent with a diagnosis of ITP. Minimal response to steroids, both standard and high dose, prompted second-line therapy with thrombopoietin receptor agonists (TPO RAs; Romiplostim, later switched over to Eltrombopag), which produced no significant response. Immunomodulation with Sirolimus was tried next with no response. Due to persistent low platelet counts and substantial bleeding on two separate occasions occurring later during her hospitalization (an episode of gross hematuria and another of lethargy with central nervous system microbleeds on neuroimaging), multiple platelet transfusions were given on an intermittent schedule, which provided variable and short-lived responses (Supplemental Fig. 1).

Extensive workups for infectious, rheumatological, and immunological causes were negative including ANAs. Laboratory-based testing for autoimmune lymphoproliferative disorder (ALPS) and a gene panel for inherited thrombocytopenias were also negative. An ITP anti-platelet antibody test panel showed very elevated titers of anti-GP IIB/IIIA antibodies (range 101-301; cut-off positive value ≥ 5.0) (Versiti, Wisconsin). Whole exome sequencing revealed a de novo missense variant in *TLR7*, c.2453G>T that led to an amino acid change from glycine to valine (p. G818V) (Fig. 1A). This variant was confirmed by Sanger sequencing (Fig. 1B) and reported to be absent in GnomAD (v4.1.0) and other population databases. The mutated glycine residue is highly conserved (Fig. 1C), lies between the last leucine-rich repeat domain and the transmembrane domain of TLR7 (Fig. 1D) and is predicted to be damaging (Polyphen-2 score of 0.998; disease causing by MutationTester; CADD score of 24.7, predicted damaging).

To test the functional consequence of this TLR7 G818V variant, we evaluated its ability to induce NF-kB signalling after transfection into HEK Blue™ reporter cells. Expression of TLR7 G818V led to a 3-fold increase in NF-κB/AP-1 activation compared with wild-type. This enhanced stimulation occurred in the absence of exogenous ligands, suggesting that the G818V substitution confers constitutive TLR7 activity (Fig. 1E). Consistent with this, Western Blot analysis of the patient’s PBMCs revealed increased amount of the approximately 65-kDa N-terminal cleaved TLR7 fragment that is produced upon TLR7 activation (Fig. 1F). To obtain further evidence of increased TLR7 activity in primary cells, expression of interferon stimulated genes (ISGs) was assessed in the patient’s PBMCs. Flow cytometry analysis revealed increased expression of the products of interferon stimulated genes (ISG) ISG15 and CD169 in blood monocytes (Fig. 1G). She also had an elevated PBMC transcriptional IFN score (z score= 373.75; cut off > 65).

Due to a continued lack of response to previous treatment, Rituximab (375 mg/m2/dose, 4 weekly doses) was started. Due to elevated IFN activity and previous reports of IFN activity in the setting of TLR7 GOF [2–4], a trial of JAK inhibition with baricitinib at a dose of 2 mg three times a day was also added. Three months after initial diagnosis, our patient achieved remission and normalized her platelet count on a combination of Rituximab (4 doses); Eltrombopag (a TPO-RA), and Baricitinib (Supplemental Fig. 1), and her anti-platelet antibodies became undetectable. 16 months after her presentation, our patient remains on treatment with Baricitinib and Eltrombopag. Her platelet counts remained stable for 7 months post- Rituximab however she had two recent drops in the past 4 months requiring intervention. Her platelet count initially dropped after a recent COVID-19 infection with full recovery after IVIG x 1 dose (1 gram/m2) and a short course of oral steroids. Over the past few weeks, her platelet count has been declining again with recurrence of bruising which corresponded to full B cell reconstitution. Thus, re-treatment with 4 weekly doses of Rituximab to deplete her B cells has been instituted. Post completion of Rituximab she will proceed to an allogeneic matched unrelated bone marrow transplant from a 10/10 HLA matched donor. In careful follow-up, our patient has no other signs of autoimmunity, lupus, or neuroinflammation. She continues to meet all developmental milestones and is following closely with neurology.

This is the third report of a child with a de novo GOF TLR7 variant initially presenting with ITP, and the first report in which severe refractory ITP remains the sole disease manifestation. The other two probands- a 1-year-old girl with a TLR7 L528I variant [3] and a 4-year-old girl with a Y264H-variant [2] – were found to have SLE or SLE-like disease. In all cases, ITP was refractory to multiple therapies. It is possible that our patient may develop systemic autoimmunity over time, and she will be monitored for this closely. The exact mechanism for development of ITP in the context of TLR7 GOF is not yet known. Enhanced TLR7 signaling in animal models has been shown to drive expansion of autoreactive immature B cells in both the follicular and extrafollicular compartments, with the latter predominantly contributing to the development of an SLE phenotype that often includes ITP [1,2,5]. In the case of the child described here, TLR7 does not require ligand binding to be activated. Such low or absent threshold for TLR7 activation is likely to activate TLR7 expressing cells like platelets, and this alone may lead to thrombocytopenia. It is also possible that TLR7-mediated platelet activation contributes to an early and specific break in tolerance to platelet antigens. Future studies may investigate whether these processes cause platelet proteins to be seen as neoantigens leading to production of high titers of anti-platelet antibodies as seen in this patient.

Together, these findings indicate that increased TLR7 signaling causes early-onset severe refractory ITP that requires combination therapy including B cell depletion and either a JAK-inhibitor or an mTOR inhibitor. Next-generation gene panel sequencing for primary immune dysregulation or whole exome testing should be strongly considered in patients with early-onset refractory ITP as the findings can have important treatment implications.

## Supplementary Material

Supplemental Fig. 1

## Figures and Tables

**Figure 1:**
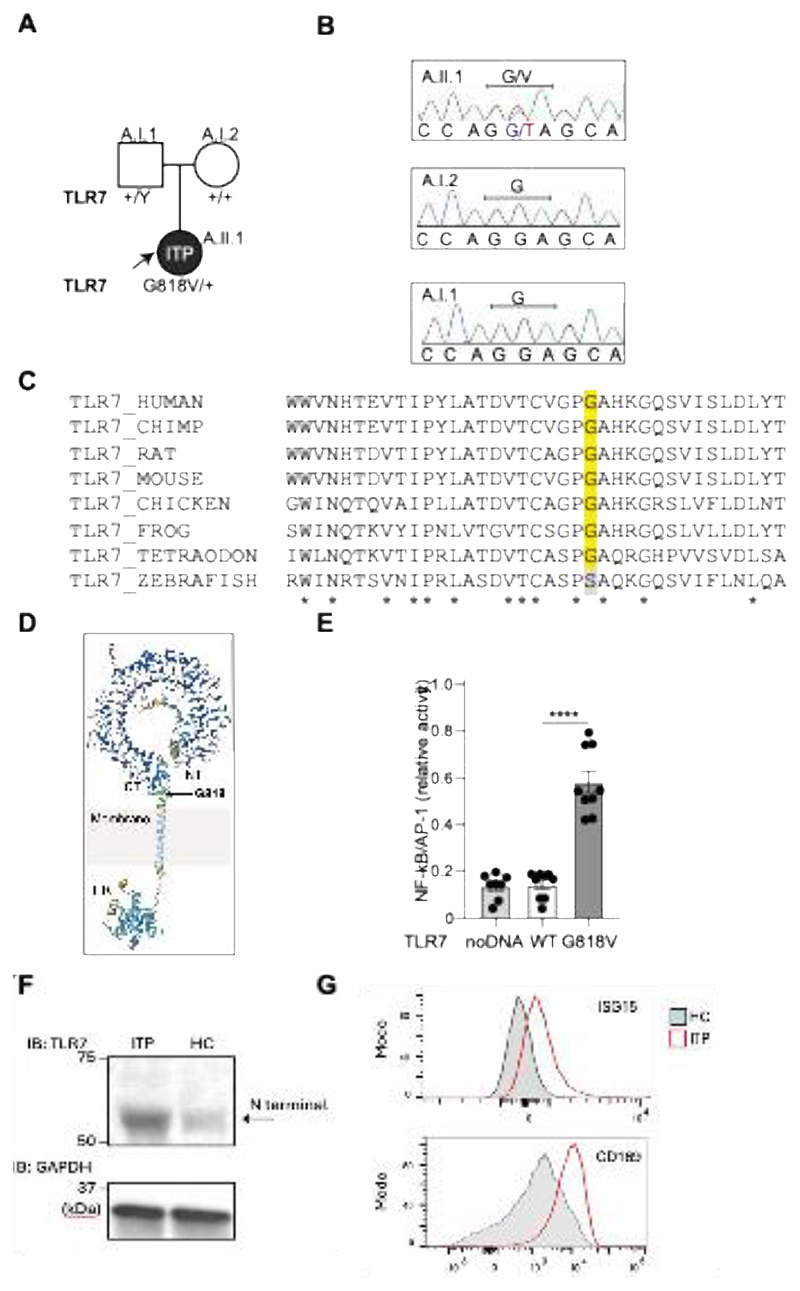
*TLR7* G818V confers gain of TLR7 function. A) Family tree of the patient presenting a heterozygous TLR-7^G818V^ de novo variant. B) Sanger sequencing results show the de novo variant (G>T) identified in the proband (A.II 1). C) Conservation of G818. D) G818 amino acid localization of TLR7 in the 3D structure as predicted by Alpha Fold. E) NF-kB/AP-1 relative activity measured in the supernatant of HEK-BlueTM cells transfected with either no DNA, wild-type TLR7 or G181V TLR7 plasmids. Data are mean and s.d. for n=9 biological replicates or transfections shown as individual dots. Statistical significance (p<0.0001) was calculated using the unpaired t-test. F) Western blot showing relative abundance of the cleaved N-terminal fragment of TLR7 in PBMC lysates from the patient (ITP) and healthy control (HC) over GAPDH, images obtained using BioRad ChemiDoc imaging system and analysed using ImageJ2 software. G) Histograms showing MFI of ISG proteins ISG15 and CD169 (Siglec-1) in monocytes isolated from the patient (ITP) and a healthy control (HC); acquired on Cytek Aurora Cytometer and analysed using FlowJo Software.
