# Supplementary material for: Refractory autoimmune thrombocytopenia in an infant with a de novo TLR7 gain-of-function variant
Source: J Clin Immunol. Author manuscript; Available in PMC 2024 Nov 18. (PMC7616824; doi:10.1007/s10875-024-01824-4)
Supplement: Supplemental Fig. 1 [file EMS200041-supplement-Supplemental_Fig__1.docx]

**Supplemental Material Content**

Supplemental Clinical Data

Supplemental Figure S1: Patient’s platelet count and corresponding treatments

Supplemental Methods

**Supplemental Clinical Data**

Lymphocyte subset panel (at diagnosis, pre-treatment):

| CD3% (Total T cells) | 71 cells/uL |
| --- | --- |
| CD3+ CD4+ % Helper T cells | 52% |
| CD3+ CD8+ % T Cytotoxic cells | 16% |
| CD19+ % B Cells | 23% |
| CD16+ CD56+ NK cells | 5 cells/uL |
| CD45+% Lymphocytes | 99% |
| CD4/CD8 Ratio | 3.2 |
| CD3 Total T cells | 6,532 (H) Reference Range 1,900- 5,900 cells/uL |
| CD3+ CD4+ T Helper Cells | 4,812 (H) Reference Range 1,400- 4,300 cells/uL |
| CD3+ CD8+ T Cytotoxic Cells | 1,520 cells/uL |
| CD19+ B Cells | 2,142 cells/uL |
| CD16+ CD56+ NK Cells | 435 cells/uL |

Immunoglobulin panel (at diagnosis, pre-treatment):

| IGA, serum | 18 mg/dL |
| --- | --- |
| IGG, serum | 1,206 mg/dL |
| IGM, serum | 59 mg/dL |

Initial anemia work up:

| Haptoglobin | 131 mg/dL |
| --- | --- |
| Lactate Dehydrogenase | 338 U/L |
| Directed AHG test | Negative |
| Reticulocyte Count | 2.61% |
| Iron | 74 ug/dL |
| Ferritin | 133 ng/mL |
| Indirect Bilirubin | 0.1 mg/dL |
| Urine Analysis | Negative for RBC’s |
| Adams T13 Activity | 91% |

Viral studies (serum):

| Adenovirus PCR | Negative |
| --- | --- |
| Epstein- Barr virus PCR | Negative |
| Parvovirus PCR | Negative |
| Hepatitis C | Negative |
| Hepatitis B | Negative |

Rheumatologic Testing:

| c4 | 17.1 mg/dL |
| --- | --- |
| c3 | 105.0 mg/dL |
| c2 | 2.1 mg/dL |
| ANA | <1:80 |
| DS DNA | <1:10 |
| CXCL9 | 497 pg/mL |
| IL-2 | 1994.1 (H) ​​ Reference Range: 175.3- 858.2 pg/mL |

**Supplementary Figure 1**

**Fig** **S1:** Platelet count (x10^3^/uL) from presentation through day 130. Frequent and sometimes continuous platelet transfusions were given until day 83 (transfusion threshold 25 x10^3^ platelets/uL), after which the platelet count had sustained recovery for several months.

**Supplemental Methods**

Study Approval

This project complied with all ethical standards set forth by the institution (Weill Cornell Medicine) as well as the Declaration of Helsinki. Written informed consent was obtained and from the patient’s parents and healthy controls per IRB approved protocol. Additional aspects of this project were determined by the Weill Cornell Medicine Institutional Review Board not to constitute human subjects research per the Code of Federal Regulations on the Protection of Human Subjects (45 CFR 46, 21 CFR 56).

Next Generation Sequencing

Whole exome sequencing (WES) was performed by Invitae Corporation, San Francisco, California and analyzed by Invitae using a panel for inherited thrombocytopenias and primary immune deficiencies. Paternity was confirmed by exome/genome sequencing trio testing, by GeneDx, Gaithersburg, MD.

Generation of human TLR7 and TLR7 mutant cell lines

HumanTLR7 wild-type and Gly818Val variants were cloned into mammalian pEZ-M07 plasmid under the CMV promoter (GeneCopoeia). HEK-Blue Null-v reporter cells containing the SEAP (secreted embryonic alkaline phophatase) reporter gene under the control of the minimal inducible IFN-b promoter fused to 5 NF-kB/AP-1 binding sites (InvivoGen) were grown in DMEM 10%heat inactivated FCS media containing 100U/ml penicillin and100mg/ml streptomycin to 70% confluency. On the day of transfection, adherent cells were washed with PBS and opti-MEM 10%FCS minimum media without antibiotics was added. HEK-Blue cells were transfected with FuGENE HD transfection reagent to DNA ratio 4:1 according to manufacturer instructions (Promega- E2311). 24h later cells were harvested and plated at 200k in 48 well plate in 400ul. 48h after transfection 100ul of supernatant was harvested and mixed with HEK-Blue detection media 1:100 dilution in 500ml total volume. The hydrolysis of the substrate by SEAP produces a purple/blue colour that can be easily detected with the naked eye or quantitatively measured with a spectrophotometer (Tecan) at OD 640 nm.

Human PBMC preparation

Blood samples were collected in acid citrate dextrose tubes for purification of polymorphonuclear blood mononuclear cells (PBMCs). PBMCs were isolated by density gradient centrifugation using Ficoll density gradient medium (GE Healthcare BioSciences), frozen in fetal calf serum with 10% DMSO, and stored in liquid nitrogen.

Anti-Platelet Antibody Testing

Tests for platelet antibodies were performed by the Platelet & Neutrophil Immunology Lab at Versiti, Milwaukee, WI. The platelet antibody bead array (PABA) test was performed to identify the specific platelet glycoproteins (GP) platelet antibodies targeted [Supplem. Ref 1]. Briefly, patient’s serum was incubated with polystyrene micro-beads coated with GPIIb/IIIa, GPIa/IIa, GPIb/IX, GPIV and Class I HLA specific monoclonal antibodies. PLTs incubated with the subject serum were washed and lysed in detergent. The lysates were incubated with a mixture of the aforementioned beads to specifically capture antigen-antibody complexes. The beads were then washed and incubated with phycoerythrin (PE)-labeled anti-human IgG and PE fluorescence detected by Luminex.

Interferon Gene Scoring

Whole blood interferon gene score was performed by the Diagnostic Immunology Laboratory at Cincinnati Children’s Hospital in Cincinnati, OH. The interferon gene score was obtained using a NanoString assay (NanoString Technology, Seattle, WA) and calculation of z-scores as previously described [Supplem. Ref 2]. Briefly, blood samples were collected in PAXgene tubes (Qiagen, Germantown, MD) and total RNA was extracted. Probes corresponding to 28 Interferon response genes were included in the NanoString codeset and the nCounter Analysis System was used to quantify gene expression. Gene expression levels were normalized to control genes and healthy controls and a z-score was calculated.

Flow Cytometry

Cryopreserved PBMCs were thawed in PBS and washed once in cold PBS prior to undergoing fixable viability staining (LIVE/DEAD Fixable Aqua Dead Cell Stain Kit, Invitrogen). Cells were then stained in FACS buffer (PBS with 2% FBS, 0.2% EDTA) with surface antibodies for 30 minutes at 4°C in the dark. Surface antibodies used: CD3-BV650 (BD Biosciences), CD11c-V450 (BD Biosciences), CD14-BV605 (Biolegend), CD16-BUV496 (BD Biosciences), CD19-PE/DyLight 594 (Sysmex), CD20-BV421 (Biolegend), CD123-BV785 (Biolegend), CD169-PE-Cy7 (Biolegend), HLADR-AlexaFluor700 (Biolegend). Cell fixation and permeabilization was performed using the eBioscience FoxP3/ Transcription Factor Staining Buffer Set (Invitrogen) per manufacturer instructions. Cells were stained intracellularly with ISG15-PE (R&D Systems) in permeabilization buffer for 30 minutes at 4°C in the dark prior to washing and resuspension in FACS buffer. Cells were passed through a 70-μm ﬁlter and data were acquired on a Cytek Aurora ﬂow cytometer. Data were analyzed using FlowJo software version 10.7.

Western Blotting

Cells lysates were obtained using RIPA Lysis and Extraction Buffer (Thermo Scientific) and protein content was quantified using the BCA protein assay (Pierce). 20ug of protein prepared in reducing buffer (Pierce Lane Marker Reducing Sample Buffer) was run in each lane of a protein electrophoresis gel (BioRad, 10% Mini-PROTEAN TGX precast gels). Proteins were transferred to PVDF membranes and blocked with 5% milk for 90 minutes. After blocking, membranes were probed with TLR7 rabbit mAb (Cell Signaling Technology), 1:1000 in TSBT 1% BSA overnight at 4°C. After washing, membranes were incubated with secondary anti-rabbit IgG antibodies conjugated to HRP (1:1000 in TBST 1% BSA) for 1h at room temperature. Washes were carried out with TBST 1% BSA. West Femto SuperSignal chemiluminescent reagent (Thermo Scientific) was used for detection and images were be obtained using ChemiDoc software. Membranes were stripped using Restore stripping buffer (Thermo Scientific) prior to blocking and re-probing with rabbit GAPDH Ab (1:1000) using the above protocol. Images were analyzed using ImageJ2 software.

Statistical Analysis

NF-kB/AP-1 expression was analyzed using means and standard deviations for n=9 biological replicates or transfections. Statistical significance was calculated using the unpaired t-test. Data was analyzed and graphed using Prism version 10.0.0 (GraphPad Software, Boston, Massachusetts USA, www.graphpad.com).

**Supplemental References**

1. Metzner K, Bauer J, Ponzi H, Ujcich A, Curtis BR. Detection and identification of platelet antibodies using a sensitive multiplex assay system - Platelet Antibody Bead Array (PABA). Transfusion 2017; Jul;57(7):1724-1733.
2. Kim H, de Jesus AA, Brooks SR, et al. Development of a Validated Interferon Score Using NanoString Technology. J Interferon Cytokine Res. 2018;38(4):171-185. doi:10.1089/jir.2017.0127
